# CAREGIVING OUTCOMES AMONG INFORMAL CAREGIVERS OF PERSONS WITH MULTIMORBIDITY AND DEMENTIA: A SCOPING REVIEW

**DOI:** 10.1093/geroni/igae098.2739

**Published:** 2024-12-31

**Authors:** Ruotong Liu, Katherine D Peak, Em Trubits, Ana Quiñones

**Affiliations:** Oregon Health & Science University, Portland, Oregon, United States; Oregon Health & Science University, Portland, Oregon, United States; Portland State University, Portland, Oregon, United States; Oregon Health & Science University, Portland, Oregon, United States

## Abstract

An emerging field of research involves caregiving among older adults with Alzheimer’s disease and related dementia (ADRD) and co-occurring chronic conditions (ADRD-multimorbidity). This scoping review aims to assess, synthesize, and identify gaps in the body of literature on caregiving outcomes among informal caregivers of individuals with ADRD-multimorbidity. We adhered to the scoping review framework by Arksey and O’Malley (2005) and Levac and colleagues (2010), which encompassed five steps: 1) identify the research question(s), delineate the inclusion and exclusion criteria, 2) search for relevant studies, 3) select studies, 4) chart the data, and 5) collate, summarize, and report results. Electronic databases including Ovid Medline, CINAHL, Embase, and PsycINFO were searched to identify relevant studies. A total of 1856 articles were identified and 29 were included in the final review. The majority of studies were quantitative, cross-sectional studies. The two most commonly examined caregiving outcomes were caregiver burden and psychological wellbeing. Most research on caregiver outcomes treated care recipients’ cognitive impairment and chronic conditions separately, rather than exploring their interaction. Most studies examining caregiver burden utilized Zarit Burden Index and its variants. Specific psychological wellbeing outcomes displayed great variability across studies. Despite challenges in synthesizing the extensive variability in the way cognitive status, ADRD-multimorbidity, and caregiving outcomes were measured, included, and reported, this review underscores the intricate challenges of caregiving, especially when dealing with both cognitive impairments and co-occurring chronic conditions. This complexity underscores the need for a deeper understanding of the diverse needs facing caregivers of people with ADRD-multimorbidity.

